# How to navigate the application of ethics norms in global health research: reflections based on qualitative research conducted with people with disabilities in Uganda

**DOI:** 10.1186/s12910-021-00710-7

**Published:** 2021-10-18

**Authors:** Muriel Mac-Seing, Louise Ringuette, Kate Zinszer, Béatrice Godard, Christina Zarowsky

**Affiliations:** 1 Department of Social and Preventive Medicine, School of Public Health, Université de Montréal, Montreal, Canada; 2 Centre de recherche en santé publique, Université de Montréal et CIUSSS du Centre-Sud-de-l’Île-de-Montréal, Montreal, Canada; 3 Laboratoire Transformation Numérique en Santé, Montreal, Canada; 4 School of Public Health, University of the Western Cape, Cape Town, South Africa

**Keywords:** Global health research, Research ethics norms, Ethics in practice, Privacy, Availability and management of financial resources, Disability, Uganda

## Abstract

**Background:**

As Canadian global health researchers who conducted a qualitative study with adults with and without disabilities in Uganda, we obtained ethics approval from four institutional research ethics boards (two in Canada and two in Uganda). In Canada, research ethics boards and researchers follow the research ethics norms of the *Tri-Council Policy Statement: Ethical Conduct for Research Involving Humans* (TCPS2), and the *National Guidelines for Research Involving Humans as Research Participants* of Uganda (NGRU) in Uganda. The preparation and implementation of this qualitative research raised specific ethical issues related to research participant privacy and the importance of availability and management of financial resources.

**Main body:**

Our field experience highlights three main issues for reflection. First, we demonstrate that, in a global health research context, methodological and logistic adjustments were necessary throughout the research implementation process to ensure the protection of study participants’ privacy, especially that of people with disabilities, despite having followed the prescribed Canadian and Ugandan ethics norms. Data collection and management plans were adapted iteratively based on local realities. Second, securing financial support as a key aspect of financial management was critical to ensure privacy through disability-sensitive data collection strategies. Without adequate funding, the recruitment of research participants based on disability type, sex, and region or the hiring of local sign language interpreters would not have been possible. Third, although the TCPS2 and NGRU underscore the significance of participants’ privacy, none of these normative documents clearly express this issue in the context of global health research and disability, nor broadly discuss the ethical issue related to financial availability and management.

**Conclusions:**

Conducting research in resource limited settings and with study participants with different needs calls for a nuanced and respectful implementation of research ethics in a global health context. We recommend a greater integration in both the TCPS2 and NGRU of global health research, disability, and responsible conduct of research. This integration should also be accompanied by adequate training which can further guide researchers, be they senior, junior, or students, and funding agencies.

## Background

In Canada, researchers, be they senior, junior or students, and research ethics board (REBs) must follow the research ethics norms of the *Tri-Council Policy Statement: Ethical Conduct for Research Involving Humans* (TCPS2) [1, 2], when addressing the expected ethical issues for a research project evaluation. Following this normative document is required by the Canadian research granting agencies, namely the Canadian Institutes of Health Research, Research Natural Sciences and Engineering Research Council of Canada and Social Sciences and Humanities Research Council, in order to receive and administer research project funds. When a research project is undertaken in another country, which is often the case in global health research, researchers need to further secure ethics approval from in-country REBs at national and/or regional levels [3]. Specific ethical issues emerge from global health research, such as resource limitations, population vulnerability, lack of human rights protection, and in relation to the status of researchers when they are doctoral and postdoctoral fellows [4]. Studies have also highlighted the importance of understanding the practical realities of applying ethical principles and norms in “real world contexts” such as in Africa to optimise health research collaboration [5].

We are Canadian researchers residing in the province of Québec who conducted a qualitative study in Uganda, from November 2017 to the end of April 2018. The qualitative research, reported in detail elsewhere [6, 7], was part of a broader mixed methods project which examined the relationships among legislation, health policy and the utilisation of sexual and reproductive health (SRH) services by people with disabilities in Uganda. The qualitative research included in-depth semi-structured interviews (n = 45) with people with different types of disabilities (physical, vision, hearing, mental, and intellectual), national organisations and decision-makers, focus groups (n = 9) with healthcare providers, disabled people’s organisations, and people with disabilities, as well as non-participant observations of health facilities (n = 7), in three northern districts (Gulu, Amuru, and Omoro) and Kampala, the capital of Uganda.

We sought ethics approval from our Canadian institutional REB, which followed the principles and ethics norms of the TCPS2 [1]. Based on three core principles—respect for persons, concern for welfare, and justice (TCPS2, article 1.1)—the TCPS2 recommends a proportional approach when evaluating projects, which considers the vulnerability of study participants, such as pregnant women, people with disabilities, and minors, and the risks related to the implementation of any research project [1]. We sought two additional ethics approvals from the Uganda national and regional REBs, which followed the principles and norms stipulated in the *National Guidelines for Research Involving Humans as Research Participants* of Uganda (NGRU) [8]. The NGRU provide mechanisms to protect the rights and welfare of research participants, promote ethical standards and procedures, and ensure that researchers consider the social and cultural values of participating communities [8].

In the preparation phase in Canada and after, we diligently responded to REBs’ request forms and received clearance from the *Centre de recherche du Centre hospitalier de l’Université de Montréal* (CR-CHUM) (17.127-CÉR, 1 August 2017); the Research Ethics Committee in Sciences and Health of the Université de Montréal (CERSES-20-074-D, 13 May 2020), following a change of research affiliation in Canada; the Lacor Hospital Institutional and Research Ethics Committee (LHIREC - 019/07/2017); and the Uganda National Council for Science and Technology (SS-4451, 14 November 2017). Throughout the study implementation process in Uganda, we constantly attempted to address and critically reflected on how we were applying the prescribed ethics norms in a “real world” context. We were confronted with two main ethical issues related to privacy and another important issue related to the availability and management of financial resources. These issues assume a heightened significance for the following reasons: (1) respect for privacy is a key ethical issue in both TCPS2 and NGRU, and is a fundamental right in Québec [9], Canada [10], Uganda [11] and internationally [12]; and (2) securing adequate financial support and managing finances responsibly were crucial to being able to deploy various strategies to optimise the research including respecting research participants’ privacy, particularly for people with different disabilities, and respecting the principle of justice as set out by the TCPS2 [2] and other international standards such as the International Ethical Guidelines for Health-related Research Involving Humans [13].

Privacy encompasses three concepts: privacy, confidentiality, and protection of personal data. Privacy specifically refers to “[…] the right to be left alone” [14], and “an individual’s right to be free from intrusion or interference by others” [1]. In the TCPS2, confidentiality is defined as an “ethical and/or legal responsibility of individuals or organizations to safeguard information entrusted to them, from unauthorized access, use, disclosure, modification, loss or theft” [1]. In the Québec legislation on the protection of personal information [15], privacy mainly refers to the protection of personal data such as those collected during and after study implementation. In Uganda, privacy and confidentiality are mentioned in the NGRU but are not defined [8]. In the 2019 Uganda Data Protection and Privacy Act, privacy is further understood among a list of principles which stipulate that people who collect, process, hold or use personal data “shall be accountable to the data subject for data collected, processed, held or used” and “observe security safeguards in respect of the data” [11].

Since neither the TCPS2 nor NGRU specifically address ethical issues related to finances and their management, financial management is understood according to the Canadian and Québec policies on responsible conduct of research. Based on the Canadian policy, “Researchers are responsible for using grant or award funds in accordance with the policies of the Agencies […] and for providing true, complete and accurate information on documentation for expenditures from grant or award accounts” [16] (p.4). In the Québec policy, “Individuals and organizations at all levels should ensure the responsible allocation and management of research funds in accordance with sound academic and financial principles. This includes ensuring an efficient use of resources” [17] (p. 12).

Guillemin and Gillam argue that tensions between “procedural ethics” (which govern a rigorous process in research) and “ethics in practice” (how it is actually implemented on the ground) are revealed through the practice of reflexivity when conducting qualitative research with the participation of humans [18]. We wish to share our reflections regarding the application of ethics norms to global health qualitative research conducted with people with disabilities in Uganda, with an emphasis on participants’ privacy and the availability and management of finances.

## Main text

### Management of participant privacy

Prior to leaving for Uganda, we received ethics approval from REBs. The study protocol contained information on data collection and analysis, interview recordings and transcriptions, and the duration of data and research records storage. In the consent forms, the importance of confidentiality and privacy was underscored for people with disabilities and other study participants. To promote the understanding of participants with disabilities, the language in the consent form was simplified, pictogrammes were added, and the content was translated in Luo/Acholi (local languages). Research participants were also informed that the collected information would be kept confidential and protected from any unauthorised disclosure or damage. The term ‘anonymity’ was used to further inform participants about the storage of recordings and depersonalised transcripts in a safe and locked facility, until their destruction would occur after a period of 10 years, as requested by the first Canadian REB (CR-CHUM).

Once in the data collection phase in Uganda, in accordance with the ethical principles of the respect for persons, their dignity and autonomy, all participants were solicited to give their informed consent to participate in the study [1, 8] in the language of their preference, either in Luo/Acholi, sign language or English. They were informed about the voluntarily aspect of their consent, of the possibility to withdraw from the study at any time, and about the confidentiality of their personal data. When interviewing people with disabilities at their home or the health facility, it was ensured that they were alone with the research team. However, when it was not feasible to find a private and closed space, we were sometimes seen from afar, under a mango tree in the garden or at the back of a health facility compound improvising a quiet space with two or three chairs. While the scene might look bucolic, confidentiality was sub-optimal. Moreover, when we conducted interviews with people with hearing impairments, sign language interpreters were hired to ensure adequate understanding among and communication with people with hearing impairments. Given that sign language could be seen from afar from other people who knew about sign language (closed spaces were not always available in villages where research was conducted), the research team deployed additional care to preserve privacy and confidentiality, such as arranging the physical placement of the interpreter and study participants such that they were less visible to other people. We had to adapt to the local realities related to the lack of private spaces. In and of itself, this was not a problem for several participants with disabilities who suggested to be interviewed outside and who felt comfortable with this alternative. Interviews with national actors such as policy-makers were held privately in their office. All focus groups were also conducted in separate rooms, either in a health facility examination room or in the premise of a local disabled people’s organisation. For participants with hearing disabilities (7/32 people interviewed individually, and 6 people of two focus groups), we hired local sign language interpreters. Given the double linguistic barriers of the researcher MMS not knowing either the Ugandan sign language or Luo/Acholi, hiring sign language interpreters was necessary to communicate with and promote the autonomy of participants who were deaf and who wanted to express their own experience. To ensure confidentiality, research assistants who were recruited for a five-month period were requested to sign a confidentiality clause in their contract, while sign language interpreters, who were hired to provide an occasional service, verbally agreed to honour participants’ confidentiality.

Concerning data storage, we stated in the consent forms that data transcripts would be kept for a period of 10 years although we are not certain how this would be managed. Upon completion of data collection and before leaving Uganda, the regional Ugandan REB requested to keep the original of all consent forms in boxes stored in the protected administrative department of the hospital which partnered with MMS for this qualitative research, while the Canadian REB (at the CR-CHUM during the qualitative study implementation) also requested the same. Contrarily to what was excepted from the Canadian REB, the personal information contained in consent forms were not kept in “locked offices” in Canada but were securely kept in a hospital research/administrative department, only accessible to authorised personnel and people who sought permission to have access to this area. Consequently, MMS made photocopies of the original consent forms and brought them back to Canada to partially fulfill the Canadian REB requirement. The forms are stored at MMS home given a lack of access to locked cabinets provided by the Université de Montréal. Given that the original consent forms are in Uganda, it would be difficult to monitor whether they are currently still there and will be kept safely for a period of 10 years, as requested. Another issue related to data storage was the lack of clarity in how to safely store the interview and focus group recordings and transcriptions. Passwords were assigned to the recordings and transcriptions, with minimal access given, and the recordings and transcriptions were saved on an online institutional cloud (of the Université of Montréal), that requires a two-level authentication. The three Canadian funding agencies conducted an online consultation on the development of a policy on research data management in September 2018 [19]. The consultation findings also emphasised a lack of guidance from the TCPS2, coupled with a confusion among respondents on whether the researchers or research institution should be responsible for personal data management [20].

### Research financing and its management

Securing the necessary funds to implement field research activities in Uganda was pivotal to ensuring many ethics norms, particularly related to the inclusion of the diverse voices of women and men with different types of disabilities. Obtaining the appropriate funds enabled us to recruit sign language interpreters and two research assistants who also acted as translators. We also travelled on *boda-boda* (moto-taxi) to villages to meet and interview study participants or discuss with study stakeholders, as these villages are often inaccessible for larger vehicles. The deliberate decision to conduct these activities had concrete logistical and ethical impacts: (1) people with and without disabilities who did not speak English (one of the official languages in Uganda) and those who used the Uganda sign language (also listed among one of the official languages) were included and participated in the research; and (2) we did not restrict the interviews and focus groups to three district headquarters (which were the most ‘accessible’) or in the Ugandan capital—we were able to invest in efforts to reach more remote individuals over the course of several weeks. Without the proper financial resources, none of these accommodations would have been possible and we would have been unable to fully respect the principle of justice as described in the TCPS2 [1], international ethical norms [13], and the literature [21]. The principle of justice refers to “the obligation to treat people fairly and equitably… The recruitment process is an important component of the fair and equitable conduct of research” [2]. Specifically, this meant to recruit people with different impairments without excluding anyone on the premise that they are inapt to answer on their own [22]. As a result, this recruitment decision also required that adequate funding be available to reach people with physical, sensory, and cognitive impairments directly in their village or to be able to bring them to the interview point, such as hiring *boda-boda* drivers and paying for their fuel, hiring sign language interpreters, or paying for the transport for the guide of people with vision impairments. These research decisions are important as they have a direct impact on the inclusion or exclusion of research participants with disabilities. However, decisions on how to spend research funding, such as on inclusion, and on how to practice responsible management of finances are not explicitly stated in TCPS2 or NRGU. Neither the NGRU nor the TCPS2, both of which have a section on conflicts of interest, address research financing and management of funds. This specific aspect is rather addressed in the *Canadian Tri-Agency Framework on Responsible Conduct of Research* [16] and the *Policy for the Responsible Conduct of Research* of the *Fonds de recherche du Québec*, the provincial research funding agency. Hence, a better alignment and connection between all these guiding and normative documents would be useful for researchers.

### How can ethics norms be better addressed when conducting global health research with people with disabilities?

It is important to note that several of the methodological and logistic adaptations made throughout the qualitative study implementation were based on MMS’ experience of working for several years with people with disabilities in sub-Saharan Africa. These accommodations included simplifying the language in the consent form coupled with the use of pictogrammes, hiring sign language interpreters, and budgeting for these activities accordingly to ensure disability-sensitive data collection and inclusion of people with different types of disabilities. This prior knowledge of the communication and accessibility needs of people with disabilities was required in order to appropriately address certain ‘real world’ ethical issues, which would not have been possible by simply following the broad principles and ethics norms of privacy stipulated in normative documents and REBs’ official requirements. Historically, people with disabilities, those with intellectual disabilities in particular, have been either denied their rights by being included in medical experiments as “guinea pigs” or excluded from research activities due to discrimination and overprotection [23]. To reach the principles of fairness and equity in research participation [1], previous experience taught us that budget planning was necessary to reach these objectives. By doing so, it provided the opportunity to people with all types of disabilities to participate in the study, allowed them to express their own views and to be heard [24]. According to the Canadian Coalition for Global Health Research, inclusion of historically marginalised groups is a key principle for sound global health research [25]. The exclusion of a certain group is compounded by power dynamics which can be addressed by not only acknowledging marginalised groups, but by actively promoting their voices and knowledges [26]. Global health scholars advocate for more equity in global health research by embracing an epistemic positionality that further promotes the conduct of research toward social justice [27].

In light of the experiences of “ethics in practice” described above, we make three suggestions. First, we suggest a greater consideration of global health research and disability in the updated 2018 TCPS2 [2] in the same way qualitative research or research with Indigenous communities have been considered. Although global health research and ethics have been widely discussed [28–30], and disability in global health research to a lesser extent due to under-prioritisation [31], global health research and disability have not received the same attention in the TCPS2 and NGRU. Additional information on these topics can help researchers, funding agencies, and institutional REBs to better address the ethical issues of privacy in a more comprehensive manner. Literature has reported that some REBs lacked training on the full scope of what privacy entails [32]. This specific issue deserves further reflection and discussion at provincial and national levels, both in Canada and Uganda. Second, given the importance of adequate financing and how the funding is spent to achieve the research objectives and ethics norms, information on availability and management of financial resources should be made more explicit in both the TCPS2 and NGRU. Neither the TCPS2 nor NGRU devote any specific articles or chapters on the importance of and relationships between funding mobilisation and availability and logistical ability to honour ethics principles including justice. These normative documents can provide illustrative field cases on how ethics principles can be directly linked to funding mobilisation and availability, as previously mentioned. This would enable researchers to contextualise ethics norms in practice more pragmatically and prepare accordingly, beyond theory [18]. It would also help to make visible some of the ethical implications of inadequate field research funding.

Finally, we suggest that a greater consideration of global health and disability as well as responsible conduct of research in normative documents be accompanied with more training on ethical issues for global health researchers and trainees. This last recommendation is also echoed in the literature on qualitative research and the importance of privacy [33]. For example, training can be offered before or during the research protocol development, with an emphasis on legal and ethical issues related to each step of the privacy cycle (data collection, utilisation, conservation and destruction) [34]. Understanding and applying the full scope of ethics norms such as related to privacy and financial management, needs to go beyond theory and be closely linked to practice in the “real world”.

## Conclusions

Conducting research in resource limited settings and with study participants with different needs calls for a nuanced and respectful implementation of research ethics in a global health context. This implies an ongoing practice of reflexivity and addressing more proactively the potential tensions between procedural ethics and ethics in the conduct of global health qualitative research with people with disabilities [4, 6, 18]. Respecting ethics principles and ensuring that the participation of marginalised and vulnerable populations such as people with disabilities requires a research team that is sensitive to the rights of people with disabilities [6]. Beyond sensitivity, however, it requires that team members pays attention to disability-sensitive practice for research to be inclusive of and accessible to people with disabilities, such as by devising accessible research tools (e.g. consent forms with pictogrammes [6] and the use of photovoice [35]) and by carefully selecting team members. Based on praxis and the local reality in Uganda, we had to iteratively adapt our approach to respect the privacy of research participants and research objectives. More importantly, without adequate financial resources, key accommodations would not have been possible. To promote a more comprehensive understanding of ethics norms, we recommend a greater integration of global health research, disability, and responsible conduct of research in normative documents such as the TCPS2 and NGRU. This integration should also be accompanied by adequate training, such as online modules, which can further guide researchers and practitioners in how to prepare a more detailed data management plan and better understand the necessary steps to be taken to manage finances responsibly.

## Data Availability

Not applicable.

## Abbreviations

CERSES: Research Ethics Committee in Sciences and Health of the Université de Montréal
CR-CHUM: *Centre de recherche du Centre hospitalier de l’Université de Montréal*
LHIREC: Lacor Hospital Institutional and Research Ethics Committee
NGRU: National Guidelines for Research Involving Humans as Research Participants of Uganda
REB: Research Ethics Board
SRH: Sexual and Reproductive Health
TCPS2: Tri-Council Policy Statement: Ethical Conduct for Research Involving Humans
